# Reconsidering amino acid matrix values: 6-phytase benefits beyond phosphorus release but limited growth recovery in lysine-deficient diets

**DOI:** 10.1016/j.psj.2026.106496

**Published:** 2026-01-22

**Authors:** Cengizhan Mızrak, Edvin Rey, Yusuf Cufadar

**Affiliations:** a Doa Nutrition Gida & Tarim Aş, Ankara, Türkiye; b AVESA Research & Consultancy, Ankara, Türkiye; c Department of Animal Science, Feeds and Animal Nutrition, Faculty of Agriculture, Selçuk University, Konya, Türkiye

**Keywords:** Broiler, Phytase, Gut integrity, Low-digestable Lysine, Performance

## Abstract

The efficancy of commercial 6-phytases in reduced digestible lysine (**dig. Lys**) diets, formulated to the ideal amino acid (**AA**) profile, remains insufficiently studied. A total of 896 Ross 308 broilers were assigned to seven dietary treatments for 41 days (8 replicates × 16 birds). Diets included a positive control (T1; dig. Lys per Ross 308 guidelines, no phytase), a negative control (T2; 3% dig. Lys below guidelines, no phytase), and five NC diets with different 6-phytases (T3–T7). Available phosphorus (**avP**) in T1 and T2 was supplied via dicalcium phosphate, while phytase diets were formulated with matrix contributions of 0.19% calcium (**Ca**), avP, and 0.03% Na. Parameters assessed were performance, intestinal morphology, caecal short-chain fatty acids (**SCFAs**), nutrient digestibility, tibia mineralization, and microbial counts. T1 birds showed significantly higher (*p* < 0.05) BW and BWG than all others. Despite partial recovery with phytase, FCR increased (*p* < 0.05) by 3.85–5.12% in T3–T7 compared to T1. Acetate and total SCFAs increased in phytase groups (notably T4, T5), while butyrate rose in T5 and T7. T5 birds had the tallest villi, and T4–T5 recorded the largest villus surface area. Tibia ash and P were elevated in T5 and T7. Digestibility of phosphorus, crude protein (**CP**), and dry matter increased with phytase, peaking in T4–T7 (*p* < 0.05). Aerobic and lactic acid bacteria increased in T4–T7, while coliforms were unaffected. Certain phytases, especially in T4, T5, and T7, improved gut health, nutrient utilization, and bone mineralization in reduced-dig. Lys diets, though growth performance was not fully restored. In conclusion, phytase manufacturers may need to re-evaluate their recommended AA matrix values.

## Introduction

The critical role of exogenous phytase in degrading phytate to release phosphorus (**P**), along with other bound nutrients, has been extensively documented, particularly over the past two decades (Golzar Adabi et al., 2025). Numerous studies have reviewed key aspects of calcium (**Ca**) and P metabolism, including accurate estimation of nutrient requirements and methods for assessing phytate content in feed ingredients (Cowieson et al., 2016; Li et al., 2017; Zaefarian et al., 2021). More recently, Golzar Adabi et al. (2025) emphasized in their review that determining the precise levels of total P and phytate fractions in plant-based ingredients is essential for formulating Ca- and P-balanced diets and for the effective application of phytase in poultry nutrition.

One of the main antinutritional effects of phytate in monogastric animals is its ability to form complexes with dietary proteins, reducing their solubility and digestibility, while also inhibiting digestive enzymes such as proteases (Cowieson et al., 2016) which can elevate endogenous amino acid (**AA**) losses. This interaction may stimulate increased mucin secretion and disrupt intestinal AA absorption by impairing sodium (Na)-dependent transport mechanisms (Cowieson et al., 2004; Woyengo et al., 2013). Consequently, supplementing diets with phytase (specially superdosis levels) beyond P release, has been shown to enhance the absorption of other minerals including Ca, Mn, Mg, and Na and improve the digestibility of CP and AAs (Cufadar et al., 2024; Farhadi et al., 2017; Golzar Adabi et al., 2025; Liu et al., 2007; Raei et al., 2025).

However, the extent to which phytase contributes to energy and AA release remains debated. While some studies report improvements of up to 2% in apparent metabolizable energy and AA digestibility, others suggest more modest benefits, such as an increase of only 0.062 MJ ME/kg, with minimal changes in AA availability (Leeson and Summers, 2005). In broilers, Babatunde et al. (2021; 2022) demonstrated that increasing phytase supplementation from 500 to 4000 FTU/kg led to a consistent rise in apparent ileal digestibility (**AID**) of AAs across both starter and grower phases, regardless of dietary phytate levels.

Inaccurate estimation—either over- or underestimation—of these matrix values can lead to suboptimal diet formulation and potential economic losses in commercial poultry production (Bedford et al., 2016). Numerous commercial phytase products are available on the market, each accompanied by distinct AA matrix recommendations. However, the meta-analysis conducted by Cowieson et al. (2017) highlights a critical inconsistency: it is scientifically unjustified for phytases with comparable P release capacities to exhibit markedly different AA matrix values. While some variation may exist due to differences in the intermediate myo-inositol phosphate isomers produced by specific phytase types or by host and microbial phosphatases, the primary determinants of extra-phosphoric nutrient release are the degree of phytate hydrolysis occurring within the gastrointestinal tract and the intrinsic digestibility of the AAs present in the diet's basal ingredients.

Commercial enzyme manufacturers often assign matrix values for Ca, P, and AAs to their phytase products, which has fostered a competitive market where enzymes are judged based on their nutrient release potential. Although some commercial poultry nutritionists base feed formulation and enzyme selection on these matrices and associated costs, many ultimately revert to using their own empirically derived values rather than those provided by enzyme companies.

Despite extensive research, limited data are available comparing the effects of different superdosed commercial 6-phytase products in broiler diets low in digestible AAs, particularly in relation to growth performance, intestinal morphology, cecal short-chain fatty acid (**SCFA**) concentrations, gut microbiota, and ileal nutrient digestibility. Therefore, the present study was designed to explore these parameters under such dietary conditions.

## Materials and methods

### Ethical approval

The animal experiment was conducted at the Application and Research Facility of Prof. Dr. Orhan Düzgüneş at Selçuk University Agriculture Faculty / Türkiye. All animal experiments were conducted in accordance with the ethical guidelines set forth by the local ethics committee of Selçuk University. The procedures employed in this study were in compliance with the ethical principles governing the treatment of animals.

### Birds, housing, diets, and experimental design

A total of 896 one-day-old male Ross 308 broiler chicks were obtained from a local hatchery and reared on floor pens lined with wood shavings for a period of 41 days. Upon arrival, chicks were randomly allocated to seven dietary treatments. Each treatment comprised eight replicate pens containing 16 chicks per pen, yielding 128 chicks per treatment and a total of 56 pens.

Feed and water were provided ad libitum using standard feeders and nipple drinkers.

Environmental conditions were carefully controlled. The initial brooding temperature was set at 33°C for the first three days, then gradually decreased to 24°C and maintained thereafter. Lighting was scheduled for 23 hours of light and 1 hour of darkness during the first three days, transitioning to 20 hours of light and 4 hours of darkness from day 4 to day 41.

The experimental treatments consisted of: a positive control (T1), formulated according to Ross 308 nutrient guidelines (2022) without phytase; a negative control (T2), with digestible lysine (**dig. Lys**) reduced by 3% relative to Ross 308 recommendations without phytase; and five phytase-supplemented diets (T3–T7), each based on the T2 formulation and containing a different commercial 6-phytase product. All experimental diets were formulated according to the ideal amino acid ratio concept. Accordingly, reductions were applied not only to dig. Lys but also to other dig. AAs during feed formulation, while maintaining constant ratios relative to dig. Lys.

For T1 and T2, available phosphorus (avP) was supplied via dicalcium phosphate. In T3–T7, phytase contributions were considered with a matrix value of 0.19% for both Ca and avP and 0.03% for Na. Ingredients and nutrient composition of experimental diets are shown in Table 1 and the level of analysed phytase in diets are presented in Table 2.

**Table 1:** Ingredients and chemical composition of experimental diets (As fed basis)^1^.

Ingredients (kg/ton)	Starter PC	Starter NC	Starter NC + PHY	Grower PC	Grower NC	Grower NC + PHY	Finisher PC	Finisher NC	Finisher NC + PHY
Maize	483.64	501.90	520.00	540.55	555.68	573.91	553.52	575.52	594.69
Soybean meal	422.46	406.86	404.87	372.02	359.23	357.12	361.51	342.07	339.07
Soybean Oil	47.08	44.70	38.65	48.64	46.70	40.64	53.10	50.25	44.07
Dicalcium Phosphate	20.65	20.76	9.84	16.44	16.52	5.60	13.08	13.21	2.30
Limestone	10.12	10.12	11.60	7.56	7.57	9.05	6.96	6.97	8.45
DL- Methionine	3.94	3.76	3.73	3.55	3.35	3.32	3.02	2.88	2.86
L-Lysine HCL	3.35	3.25	3.26	2.89	2.71	2.73	1.88	2.01	2.05
Sodium Bicarbonatee	2.90	2.90	1.84	2.76	2.72	1.66	2.27	2.38	1.33
Salt	1.96	1.98	1.97	2.12	2.16	2.15	2.50	2.43	2.41
L-Threonine	1.73	1.62	1.61	1.43	1.38	1.37	0.84	0.88	0.88
Vitamin - Mineral Premix^2^	1.00	1.00	1.00	1.00	1.00	1.00	1.00	1.00	1.00
L-Valine	0.69	0.63	0.61	0.56	0.46	0.44	***	***	***
Choline Chloride	0.47	0.53	0.52	0.48	0.52	0.51	0.32	0.40	0.39
Phytase	***	***	0.50	***	***	0.50	***	***	0.50
Total	1000	1000	1000	1000	1000	1000	1000	1000	1000
Chemical composition^3^									
Crude Protein, %	23.37 (23.26)	22.75 (22.86)	22.78 (22.61)	21.36 (21.42)	20.84 (20.93)	20.87 (20.75)	20.76 (20.88)	20.03 (20.18)	20.03 (20.15)
Metabolisable Energy (kcal/kg)	2975	2975	2975	3050	3050	3050	3100	3100	3100
Dig. Lysine, %	1.32	1.28	1.28	1.18	1.14	1.14	1.08	1.05	1.05
Dig. Methionine, %	0.69	0.66	0.66	0.63	0.60	0.60	0.57	0.55	0.55
Dig. Met+Cys, %	1.00	0.97	0.97	0.92	0.89	0.89	0.86	0.83	0.83
Dig. Arginine, %	1.40	1.36	1.36	1.27	1.24	1.24	1.24	1.193	1.19
Dig. Threonine, %	0.88	0.85	0.85	0.79	0.77	0.77	0.72	0.70	0.70
Dig. Leucine, %	1.69	1.65	1.66	1.57	1.54	1.55	1.55	1.50	1.51
Dig. Isoleucine, %	0.88	0.86	0.86	0.80	0.78	0.78	0.78	0.75	0.75
Dig. Valine, %	1.00	0.97	0.97	0.91	0.88	0.88	0.84	0.81	0.81
Dig. Tryptophane, %	0.23	0.22	0.22	0.20	0.20	0.20	0.20	0.19	0.19
Ca, %	0.95	0.95	0.95	0.75	0.75	0.75	0.65	0.65	0.65
P available, %	0.50	0.50	0.50	0.42	0.42	0.42	0.36	0.36	0.36
Total P (analysed), %	0.52	0.53	0.33	0.44	0.43	0.25	0.37	0.38	0.19
Na, %	0.18	0.18	0.18	0.18	0.18	0.18	0.18	0.18	0.18
K, %	1.07	1.05	1.05	0.99	0.97	0.97	0.97	0.94	0.94
Cl, %	0.23	0.23	0.23	0.23	0.23	0.23	0.23	0.23	0.23
Choline, %	1700	1700	1700	1600	1600	1600	1500	1500	1500

1 PC, positive control containing recommended levels of dig. AAs without any additional exogenous enzyme, avP provided by DCP; NC, negative control, containing 3% lower dig. AA than recommendation, without any additional exogenous enzyme, avP provided by DCP; NC + PHY, containing 3% lower dig. AA than recommendation, with additional 2000 FTU/kg of different dietary commercial 6-phytase products, Ca and avP matrix included as 0.19%; and 0.03% for Na.

2 Premixes provided per kilogram of complete diet: 4.13 mg retinyl acetate (A); 125 µg cholecalciferol (D3); 100 mg dl-α-tocopheryl acetate (E); 4 mg menadione (K3); 4 mg thiamine (B1); 9 mg riboflavin (B2); 65 mg niacin (B3); 20 mg calcium d-pantothenate (B5); 4.5 mg pyridoxine (B6); 0.28 mg biotin (B7); 2.2 mg folic acid (B9); 0.02 mg vitamin B12; 16 mg copper (Cu-sulphate, CuSO4); 1.5 mg iodine (Ca-iodate, Ca(IO3)2); 25 mg iron (Fe-sulphate, FeSO4); 120 mg manganese (Mn-oxide, MnO); 0.3 mg selenium (Na-selenite, Na2SeO3); 120 mg zinc (Zn-oxide, ZnO).

3 Values given inside the parentheses indicate analysed results, and values outside the parentheses refer to calculated values.

**Table 2:** Experimental treatments and analysed phytase content (FTU/kg) of dietary treatments^1^.

Treatments	Experimental treatments	Starter	Grower	Finisher
T1	Positive control (PC; dig. Lys per Ross 308 guidelines (2022), no phytase	53	48	57
T2	Negative Control (NC; 3% dig. Lys below Ross 308 guidelines (2022), no phytase	55	62	66
T3	NC + 2000 FTU/ kg Phytase A	2020	2057	2010
T4	NC + 2000 FTU/ kg Phytase B	1995	2024	2040
T5	NC + 2000 FTU/ kg Phytase C	2055	2035	1997
T6	NC + 2000 FTU/ kg Phytase D	2047	2058	2029
T7	NC + 2000 FTU/ kg Phytase E	2065	2016	2038

1 T1, positive control containing recommended levels of dig. AAs without any additional exogenous enzyme, avP provided by DCP; T2, negative control, containing 3% lower dig. AA than recommendation, without any additional exogenous enzyme, avP provided by DCP; T3-T7, as NC but supplemented with 2000 FTU/kg of different dietary commercial 6-phytase products, with a matrix contribution of 0.19% for Ca and avP and 0.03% for Na.

To avoid potential misinterpretation, enzyme treatments in this study are not linked to specific commercial brand names. Instead, only the active substance—6-phytase produced by genetically modified strains—is reported, without reference to the treatment order used in the experiment. The phytase enzymes evaluated were derived from one of the following microorganisms: Aspergillus niger (DSM 25770), Aspergillus oryzae (DSM 33737), Trichoderma reesei (CBS 146250), Komagataella phaffii (DSM 32854), and Trichoderma reesei (CBS 126897).

Final diets were analyzed for AA composition using the method of Llames and Fontaine (1994), while total AAs were assessed according to AOAC 982.30 E (AOAC, 2006). To estimate nutrient digestibility, titanium dioxide (**TiO₂**) was added at 0.5% to all diets three days before termination of the trial (Cufadar et al., 2024; Gül et al., 2025; Raei et al., 2025).

### Growth performance

Pen based body weight gain (BWG) and feed intake (FI) were recorded on days 10, 24, and 41. Feed conversion ratio (FCR) was calculated as FI divided by BWG.

### Sample collection and laboratory analysis

On day 41, two birds per pen with body weights representative of the replicate mean were selected, leg-banded, and humanely euthanized by jugular exsanguination, followed by evisceration. All measured variables were calculated as the mean of the two birds sampled from each replicate.

Cecal contents were collected for short-chain fatty acid (SCFA) and microbial analysis (Ceylan et al., 2024; Cufadar et al., 2024; Gül et al., 2025; Raei et al., 2025). Ileal digesta from the distal third of the ileum was used to determine apparent ileal digestibility (AID) of selected nutrients. Jejunal tissue was sampled for histomorphometric evaluation, and left tibias were removed for mineral assessments (Cufadar et al., 2024; Gül et al., 2025; Raei et al., 2025).

Ileal digesta and cecal contents samples were individually collected and stored at −20°C until analysis. AID was calculated using the following equation (Cufadar et al., 2024; Gül et al., 2025; Raei et al., 2025):

$$\text{AID}\;\left(\%\right)=1-\left\{\left[\left(\text{Ti}O_{2\_}\text{feed}/\text{Ti}O_{2\_}\text{digesta}\right)\times \left(\text{Nutrient}\_\text{digesta}/\text{Nutrient}\_\text{feed}\right)\right]\right\}\times 100$$

A 5 cm segment of mid-jejunum was fixed in 10% buffered formalin. Villus surface area (VSA) was calculated as:

VSA = 2π × (villus width / 2) × villus height / 10⁶ (Ceylan et al., 2024; Cufadar et al., 2024; Gül et al., 2025; Raei et al., 2025).

### Statistical analysis

All performance response data were analyzed using a completely randomized design with 7 dietary treatments, each comprising 8 replicates of 16 birds. Laboratory analysis data were similarly assessed using a completely randomized design with 7 treatments and 8 replicates, with two samples per replicate. Statistical analysis was performed using the General Linear Model (GLM) procedure of SAS software (version 9.2). When significant treatment effects were identified (*P* < 0.05), mean comparisons were conducted using Tukey's Honest Significant Difference (HSD) test. Percentage data were subjected to an arcsine square root transformation to achieve a normal distribution (Steel and Torrie, 1960).

The statistical model used for the experiment was: Yij = μ + αi + eij

Where Yij represented response variable, µ was overall mean, αi was the effect of the treatments, and eij denoted the random error.

## Results

The final BW and BWG of birds fed on positive control diet (T1) were significantly greater than those of all other dietary treatments, with the exception of T4 (Table 3; *P* < 0.05). Although phytase inclusion enhanced performance of T2, it did not fully restore BWG or FCR to values comparable with those observed in T1 (Table 3). Dietary treatments exerted no significant influence on overall FI during 25-41 d and 1-41 d of the experimental period (Table 3; *P* > 0.05). At the end of the experimental period, the T1 group exhibited a significantly lower FCR than all other treatments (Table 3; *P* < 0.05). Moreover, birds fed on diets T2, T3, and T6 showed significantly higher FCR values (Table 3; *P* < 0.05). Despite the significant beneficial effect of dietary phytase on final FCR in T4, T5, and T7, phytase supplementation did not fully restore FCR to the level recorded in T1 (Table 3; *P* < 0.05).

**Table 3:** Effects of experimental treatments on body weight (BW), body weight gain (BWG), feed intake (FI), and feed conversion ratio (FCR) of broilers^a^.

Parameters	Treatments^1^								
	T1	T2	T3	T4	T5	T6	T7	SEM	P-value
BW, g/bird									
Initial	42.63	42.49	42.61	42.64	42.66	42.45	42.52	0.072	0.98
10 d	314.41 ^a^	284.61 ^c^	290.45 ^bc^	301.92 ^ab^	293.24 ^bc^	295.15 ^bc^	300.38 ^b^	1.652	<.0001
24 d	1195.90 ^a^	1064.80 ^c^	1088.54 ^bc^	1136.74 ^ab^	1119.95 ^bc^	1072.22 ^c^	1100.26 ^bc^	7.692	<.0001
41d	3078.54 ^a^	2879.48 ^c^	2938.61 ^b^	2977.25 ^ab^	2955.46 ^b^	2926.87 ^b^	2962.14 ^b^	11.800	0.0001
BWG, g/bird									
1-10 d	271.77 ^a^	242.11 ^c^	247.84 ^bc^	259.28 ^ab^	250.58 ^bc^	252.69 ^bc^	257.86 ^ab^	1.661	<.0001
11-24 d	881.49 ^a^	780.19 ^b^	798.10 ^b^	834.82 ^ab^	826.71 ^ab^	777.07 ^b^	799.88 ^b^	6.936	<.0001
25-41 d	1882.64	1814.68	1850.06	1840.52	1835.51	1854.65	1861.87	10.914	0.78
1-41 d	3035.91 ^a^	2836.99 ^b^	2896.00 ^b^	2934.62 ^ab^	2912.80 ^b^	2884.41 ^b^	2919.61 ^b^	11.806	0.0001
FI, g/bird									
1-10 d	313.79 ^a^	292.98 ^b^	296.77 ^b^	308.38 ^ab^	298.60 ^ab^	302.35 ^ab^	307.06 ^ab^	1.622	0.003
11-24 d	1142.52 ^a^	1042.98 ^b^	1054.23 ^b^	1097.80 ^ab^	1084.34 ^ab^	1027.39 ^b^	1049.93 ^b^	8.017	0.0004
25-41 d	2975.47	2964.30	3013.09	2958.75	2943.63	3014.78	2989.43	16.998	0.91
1-41 d	4431.78	4300.26	4364.08	4362.43	4326.56	4344.51	4346.41	15.789	0.46
FCR; FI, g/BWG, g									
1-10 d	1.155 ^b^	1.210 ^a^	1.198 ^a^	1.190 ^ab^	1.192 ^ab^	1.197 ^a^	1.191 ^ab^	0.004	0.003
11-24 d	1.296 ^c^	1.338 ^a^	1.321 ^abc^	1.315 ^abc^	1.312 ^bc^	1.322 ^ab^	1.313 ^abc^	0.003	0.0007
25-41 d	1.581 ^c^	1.634 ^a^	1.629 ^a^	1.606 ^b^	1.604 ^b^	1.625 ^a^	1.606 ^b^	0.002	<.0001
1-41 d	1.460 ^c^	1.516 ^a^	1.507 ^a^	1.486 ^b^	1.485 ^b^	1.506 ^a^	1.489 ^b^	0.003	<.0001

Means within the same row without common superscripts are significantly different (*P* < 0.05).

1 T1, positive control containing recommended levels of dig. AAs without any additional exogenous enzyme, avP provided by DCP; T2, negative control, containing 3% lower dig. AA than recommendation, without any additional exogenous enzyme, avP provided by DCP; T3-T7, as NC but supplemented with 2000 FTU/kg of different dietary commercial 6-phytase products, with a matrix contribution of 0.19% for Ca and avP and 0.03% for Na.

Despite the limited improvement in growth performance, phytase supplementation had notable positive effects on gut fermentation (Table 4) and intestinal morphology (Table 5).

**Table 4:** Effects of experimental treatments on the concentration of caecal short-chain fatty acids (SCFA) of broilers.

Parameters	Treatments^1^							SEM	P-value
	T1	T2	T3	T4	T5	T6	T7		
Acetate (mmol/L)	24.74 ^c^	26.36 ^bc^	29.83 ^bc^	36.89 ^a^	36.34 ^a^	31.57 ^ab^	32.44 ^ab^	0.774	<.0001
Propionate (mmol/L)	9.37 ^d^	9.63 ^dc^	11.66 ^bdc^	13.58 ^ab^	14.73 ^a^	12.65 ^abc^	13.82 ^ab^	0.360	<.0001
Butyrate (mmol/L)	9.18 ^b^	8.67 ^b^	11.09 ^ab^	11.40 ^ab^	12.57 ^a^	11.34 ^ab^	12.93 ^a^	0.337	0.001
Isobutyrate (mmol/L)	0.91 ^b^	1.01 ^ab^	1.10 ^ab^	1.05 ^ab^	1.08 ^ab^	1.17 ^a^	1.07 ^ab^	0.019	0.01
Valerate (mmol/L)	1.06	1.02	1.91	1.05	1.10	1.11	1.15	0.020	0.3
Isovalerate (mmol/L)	1.19	1.09	1.12	1.18	1.14	1.15	1.17	0.023	0.9
BCFA (mmol/L)	3.16	3.13	3.42	3.28	3.32	3.43	3.39	0.034	0.1
Total SCFA (mmol/L)	46.45 ^d^	47.78 ^dc^	55.99 ^bc^	65.14 ^a^	66.96 ^a^	58.98 ^ab^	62.57 ^ab^	1.217	<.0001

Means within the same row without common superscripts are significantly different (*P* < 0.05).

1 T1, positive control containing recommended levels of dig. AAs without any additional exogenous enzyme, avP provided by DCP; T2, negative control, containing 3% lower dig. AA than recommendation, without any additional exogenous enzyme, avP provided by DCP; T3-T7, as NC but supplemented with 2000 FTU/kg of different dietary commercial 6-phytase products, with a matrix contribution of 0.19% for Ca and avP and 0.03% for Na.

**Table 5:** Effects of experimental treatments on the jejunal histomorphology of broilers.

Parameters	Treatments^1^							SEM	P-value
	T1	T2	T3	T4	T5	T6	T7		
Villus height (µm)	1301.26 ^b^	1281.87 ^b^	1335.98 ^b^	1429.21 ^a^	1430.34 ^a^	1336.05 ^b^	1372.71 ^ab^	10.523	<.0001
Villus width (µm)	159.00	147.08	178.44	190.69	184.16	176.09	173.13	4.414	0.12
Crypt depth (µm)	198.90 ^a^	206.14 ^a^	151.11 ^b^	138.63 ^b^	143.80 ^b^	140.20 ^b^	160.56 ^ab^	5.267	<.0001
Villus surface area (mm^2^)	0.65 ^ab^	0.59 ^b^	0.75 ^ab^	0.85 ^a^	0.83 ^a^	0.74 ^ab^	0.75 ^ab^	0.020	0.004
Villus height/Crypt depth ratio	6.77 ^bc^	6.37 ^c^	9.19 ^ab^	10.07 ^a^	10.09 ^a^	9.93 ^a^	8.73 ^abc^	0.286	<.0001
Goblet cells numbers (n)	190.58 ^ab^	153.08 ^b^	189.53 ^ab^	208.36 ^a^	202.14 ^ab^	188.83 ^ab^	191.31 ^ab^	4.822	0.05

Means within the same row without common superscripts are significantly different (*P* < 0.05).

1 T1, positive control containing recommended levels of dig. AAs without any additional exogenous enzyme, avP provided by DCP; T2, negative control, containing 3% lower dig. AA than recommendation, without any additional exogenous enzyme, avP provided by DCP; T3-T7, as NC but supplemented with 2000 FTU/kg of different dietary commercial 6-phytase products, with a matrix contribution of 0.19% for Ca and avP and 0.03% for Na.

In birds assigned to treatments T4, T5, T6, and T7, cecal acetate concentrations were significantly elevated compared with those in T1 (*P* < 0.05), with the greatest increases detected in T4 and T5 at 49.0% and 46.8%, respectively (Table 4). Broilers fed on diets T1 and T2 exhibited the lowest cecal propionate concentrations, whereas propionate level was significantly higher in birds receiving treatments T4 through T7 (Table 4; *P* < 0.05). Regarding butyrate concentration, birds offered diets T1 and T2 showed the lowest values, while those in T3 through T7 displayed the highest butyrate levels; consequently, butyrate concentrations in T5 and T7 were increased by 36.9% and 40.8%, respectively (Table 4; *P* < 0.05). Total SCFA concentrations were also significantly increased in all phytase-supplemented treatments (T3–T7) compared with T1 (Table 4; *P* < 0.05). The greatest increases were observed in T4 (40.3%) and T5 (44.1%).

Jejunal histomorphological analysis indicated that birds receiving diets T4 and T5 exhibited statistically greater VH than those in the other treatment groups, with the exception of T7 (Table 5; *P* < 0.05). Crypt depth (CD) was statistically greater in birds fed T1 and T2 compared with the remaining treatments, except for T7 (*P* < 0.05). Villus surface area (VSA) in T1 was statistically comparable with that of all other treatments (*P* < 0.05); however, VSA significantly increased in T4 and T5 relative to T2 (*P* < 0.05). In addition, the VH:CD ratio was significantly enhanced in treatments T4 through T6 (Table 5; *P* < 0.05). Goblet cell counts in T1 were statistically similar to those observed in all other treatments (*P* < 0.05). Goblet cell numbers were significantly elevated in T4, representing a 9.3% increase compared with T1 (*P* < 0.05).

Phytase supplementation also positively affected bone mineralization and nutrient digestibility (Table 6).

**Table 6:** Effects of experimental treatments on bone development and nutrient digestibility of broilers.

Parameters			Treatments^1^						SEM	P-value
	T1	T2		T3	T4	T5	T6	T7		
Bone development										
Tibia ash (% of DM)	35.04 ^bc^	34.18 ^c^		36.78 ^abc^	37.19 ^ab^	38.04 ^a^	36.67 ^abc^	37.90 ^a^	0.290	0.0004
Tibia P (% of ash)	17.20 ^b^	17.12 ^b^		18.02 ^ab^	18.08 ^ab^	19.03 ^a^	18.49 ^ab^	18.74 ^ab^	0.169	0.008
Nutrient digestibility										
P (%)	63.24 ^ab^	62.09 ^b^		65.73 ^ab^	66.58 ^a^	67.38 ^a^	65.81 ^ab^	67.05 ^a^	0.427	0.001
CP (%)	69.57 ^c^	71.44 ^bc^		74.52 ^ab^	75.68 ^ab^	76.56 ^a^	73.81 ^abc^	76.19 ^a^	0.498	<.0001
DM (%)	72.56 ^b^	73.02 ^b^		76.81 ^ab^	78.52 ^a^	77.53 ^ab^	75.87 ^ab^	76.39 ^ab^	0.513	0.006

Means within the same row without common superscripts are significantly different (*P* < 0.05).

1 T1, positive control containing recommended levels of dig. AAs without any additional exogenous enzyme, avP provided by DCP; T2, negative control, containing 3% lower dig. AA than recommendation, without any additional exogenous enzyme, avP provided by DCP; T3-T7, as NC but supplemented with 2000 FTU/kg of different dietary commercial 6-phytase products, with a matrix contribution of 0.19% for Ca and avP and 0.03% for Na.

The greatest tibia ash content (% of DM) was recorded in treatments T5 and T7 and was significantly higher than that observed in birds fed diets T1 and T2 (*P* < 0.05). Tibia P concentration (% of ash) reached its maximum in T5, representing a 10.6% increase compared with T1 (Table 6; *P* < 0.05). Apparent ileal P digestibility significantly increased in T4 (7.2%), T5 (8.5%), and T7 (7.9%) relative to T2 (Table 6; *P* < 0.05). Crude protein (CP) digestibility was significantly greater in T5 and T7 treatments compared with T1 and T2, whereas no significant differences were detected among the remaining treatments for this parameter (*P* < 0.05). Dry matter (DM) digestibility was significantly higher in birds receiving T4 than in those fed T1 and T2 (Table 6; *P* < 0.05).

Microbial analysis of cecal digesta showed that total aerobic bacterial counts were significantly increased in treatments T4, T6, and T7 compared with T1 and T2 (*P* < 0.05). Lactic acid bacteria populations were likewise significantly greater in birds fed diets T4 through T7 than in those receiving T1 and T2 (*P* < 0.05). No significant differences were detected in coliform counts among the dietary treatments (Table 7; *P* > 0.05).

**Table 7:** Effects of experimental treatments on the caecal microflora population (log cfu/g) of broilers.

Parameters	Treatments^1^							SEM	P-value
	T1	T2	T3	T4	T5	T6	T7		
Total aerobic bacteria	6.65 ^bc^	5.96 ^c^	8.06 ^ab^	9.36 ^a^	8.16 ^ab^	8.84 ^a^	9.61 ^a^	0.237	<.0001
Lactic acid bacteria	6.99 ^b^	6.52 ^b^	8.16 ^ab^	9.08 ^a^	9.43 ^a^	8.90 ^a^	9.10 ^a^	0.210	<.0001
Coliform	7.48	6.89	7.44	6.99	7.23	7.97	7.19	0.160	0.652

Means within the same row without common superscripts are significantly different (*P* < 0.05).

1 T1, positive control containing recommended levels of dig. AAs without any additional exogenous enzyme, avP provided by DCP; T2, negative control, containing 3% lower dig. AA than recommendation, without any additional exogenous enzyme, avP provided by DCP; T3-T7, as NC but supplemented with 2000 FTU/kg of different dietary commercial 6-phytase products, with a matrix contribution of 0.19% for Ca and avP and 0.03% for Na.

## Discussion

The present study demonstrates that a 3% reduction in dietary dig. Lys and other AAs in the diet relative to the ideal AA profile ratio significantly compromises broiler growth performance, as reflected in the lower final BW and BWG observed in the negative control group (T2) compared to the positive control (T1). Lysine plays a critical role in skeletal muscle development and protein accretion, and lean tissue deposition in broilers, and its deficiency has been consistently associated with reduced growth efficiency in broilers. Lys also serves as the reference AA in the concept of ideal protein (Corzo et al., 2006; Faridi et al., 2015). These findings are in agreement with the well-established role of lysine as one of the first-limiting AAs in poultry diets, essential for supporting optimal performance (Leeson and Summers, 2005).

None of the phytase-supplemented groups fully restored BWG or FCR to the level of T1, suggesting that phytase cannot fully compensate for AA deficiencies even when it enhances nutrient release. This incomplete recovery suggests that, while phytase enhances nutrient release from phytate, it cannot entirely compensate for AA when the birds feeding by less 3% of dig.Lys and other AAs. Contrary to the results of the present study, some previous reports have shown no significant difference (Sobotik et al., 2024) or even an improvement (Bello et al., 2025) in FCR of broiler chickens when phytase was supplemented to nutrient-deficient diets with the application of full matrix values. Sobotik et al. (2024) fed the broilers on NC diet with reductions in ME by 87.8 kcal/kg, avP by 0.199%, Ca by 0.21%, CP by 0.72–1.03%, dig. Lys by 0.064–0.084%, and Na by 0.047%, supplemented with 2000 FTU/kg of phytase. Although the FCR did not differ significantly between birds receiving this diet and those on the PC, FCR was numerically elevated by 4.4% in the NC + phytase group. In the present study, birds fed NC diets supplemented with 2000 FTU/kg of various commercial phytases exhibited a numerical increase in FCR ranging from 3.85% to 5.12% compared to the positive control group. In another study, Jacob et al. (2000) showed that, supplementation with 600 FTU/kg of fungal phytase in wheat-based diets containing 216 g/kg CP during the starter phase and 170 g/kg CP during the grower phase resulted in reduced BWG and FI in broilers from day 1 to 42 post-hatch, while FCR remained unaffected.

The elevated FCR observed across all phytase-supplemented groups compared to the T1 reinforces the notion that AA deficiency, particularly Lys, can limit the efficiency of nutrient utilization despite improvements in digestibility. The partial reduction in FCR relative to T2 likely results from enhanced protein and P utilization suggest the compensatory effect of phytase as previously noted in studies evaluating phytase’s nutritional contribution (Cowieson et al., 2017; Wise et al., 2024). However, the persistent performance gap reinforces the principle that AA matrices assigned to phytase should be applied with caution. As demonstrated by Babatunde et al. (2021; 2022), even at high inclusion levels such as 4,000 FTU/kg, residual undigested metabolizable energy and protein can still be detected in the digesta. Commercial broiler producers may opt to restrict the matrix application of phytase to P, Ca and Na, while considering the beneficial effects of phytase on protein/AA and energy utilization as reflected in improved growth performance (Selle et al., 2023).

Despite these limitations in growth performance restoration, phytase supplementation had notable positive effects on gastrointestinal physiology and fermentation profiles. Birds receiving phytase, especially in treatments T4 and T5, exhibited significant increases in cecal acetate and total SCFA concentrations, while butyrate levels were notably higher in T5 and T7. SCFAs, particularly butyrate, a well-known trophic factor for intestinal epithelial cells, with enhanced gut epithelial integrity, anti-inflammatory effects that enhance nutrient absorption (Guilloteau et al., 2010). These findings are in line with recent studies showing that microbial phytase enhances fermentation by shifting gut microbial communities toward beneficial populations and increasing SCFA production. This is possibly by reducing undigested phytate and increasing fermentable substrates for beneficial microbes (Cufadar et al., 2024; Kiarie et al., 2013; Raei et al., 2025).

In the current study, consistent with improved fermentation, significant enhancements in intestinal morphology were observed. Birds fed phytase-containing diets exhibited increased VH, greater VSA, and reduced CD, especially in T4 and T5, indicate enhanced absorptive mucosal surface area and reduced enterocyte turnover. These enhancements are consistent with previous findings that phytase improves mucosal development by reducing the anti-nutritional effects of phytate and increasing mineral and nutrient availability (Cufadar et al., 2024; Moita et al., 2021; Nari et al., 2020). Additionally, the elevated goblet cell counts observed in T4 reflect enhanced mucin production, which may contribute to a stronger intestinal barrier and improved mucosal immunity (Tonetti et al., 2024).

Ceacal microbiota composition was also influenced by phytase, with increased counts of total aerobic and lactic acid bacteria. This shift in microbial composition is typically linked to a more balanced and health-promoting gut microbial ecosystem that enhances fermentative activity and overall gut functionality (Kiarie et al., 2013), while the increased presence of lactic acid bacteria specifically contributes to improved intestinal stability by reinforcing gut homeostasis and inhibiting the colonization of pathogenic microorganisms through competitive exclusion mechanisms (Babatunde et al., 2022; Kiarie et al., 2013; Moita et al., 2021; Zaefarian et al., 2021). The lack of differences in coliform populations suggests that phytase selectively promotes beneficial microbial growth without favoring potentially pathogenic bacteria.

In terms of skeletal development, tibia ash content and P concentration were highest in T5 and T7, mirroring the improvements in P digestibility seen in these groups. These findings reflect the known capacity of phytase to hydrolyze phytate–P complexes, liberating bound P, thereby enhancing the mineral bioavailability and supporting bone mineralization (Cufadar et al., 2024; Dersjant-Li et al., 2022; Farhadi et al., 2017; Moita et al., 2021; Raei et al., 2025). Tibia ash content is widely recognized as a reliable indicator of Ca and P adequacy in poultry (Karami et al., 2020). Supplementation of broiler diets with phytase at levels up to 2000 FTU/kg has been reported to enhance the structural development of the tibia, including improvements in bone strength, length, and diameter (Fernandes et al., 2019). Phosphorus bioavailability increases by hydrolysis of phytate by phytase, thereby supporting bone mineralization (Walk et al., 2014). Notably, the P and Ca requirements for maximizing tibia ash content often exceed those needed for optimal growth performance. Therefore, the elevated tibia ash concentrations observed in phytase fed birds compare to PC birds, may be attributed to the more liberation of phytate-bound P (Farhadi et al., 2017; Rai et al., 2025).

Improvements in P, CP and DM digestibility in phytase-fed birds further support the enzyme’s broader role in releasing nutrients bound to phytate not only minerals but also AAs, reducing anti-nutritional effects, and enhancing overall nutrient efficiency (Babatunde et al., 2021; Cowieson et al., 2016; Moita et al., 2021; Selle et al., 2023). Moita et al. (2021) reported that enhancements in intestinal morphology associated with increasing levels of phytase supplementation may serve as indicators of improved digestive and absorptive capacity, as well as more efficient nutrient utilization in broilers. Phytase enhances the utilization of dietary AAs by mitigating the anti-nutritional effects of phytate. Additionally, reducing dietary Ca levels may further improve protein and AA digestibility by lowering gastric pH, thereby enhancing pepsin activity and overall proteolytic efficiency (Cowieson et al., 2008; Walk et al., 2012). The effects of phytase on AA digestibility have been reported with varying consistency across studies. One contributing factor to this variability is the use of different indigestible markers—such as acid-insoluble ash, TiO₂, and chromic oxide (**Cr₂O₃**)—in digestibility assays. Although these markers are routinely used to estimate nutrient digestibility, they are associated with inherent biases called as 'marker bias'. Specifically, AIA has been reported to underestimate, whereas Cr₂O₃ may overestimate AA digestibility in feed ingredients. Such discrepancies can confound the accurate assessment of phytase efficacy (Cowieson et al., 2017). It is necessary to mentioned that, reductions in dietary CP levels influence apparent AA digestibility through opposing mechanisms. Lower CP diets often reduce digestibility coefficients due to dilution by endogenous and microbial AAs in the distal ileum. Replacing soybean meal—which has superior digestibility—with ingredients like maize or wheat can further reduce digestibility in low-CP diets. In contrast, synthetic AAs, assumed to be fully digestible, so their dietary inclusions may increase their digestibilities. However, phytate complicates this dynamic, as it can bind synthetic AAs depending on their isoelectric points; for instance, phytate in rice bran has been shown to bind Lys-hydrochloride in vitro. Phytase supplementation may alter these interactions by degrading phytate, yet its effects on AA digestibility remain inconsistent. Thus, fluctuations in AA digestibility under reduced-CP diets may reflect the combined influences of ingredient composition, phytate presence, and phytase activity (Selle et al., 2023).

## Conclusions

Based on the results of the current study it can be concluded that, despite the broad physiological benefits such as enhancing intestinal morphology, modulating gut microbial populations, improving nutrient digestibility, and supporting bone mineralization, phytase supplementation demonstrated incomplete recovery of performance parameters in diets deficient in dig.Lys compared with positive control. These results underscore the broader physiological role of phytase beyond its traditional function as a P-releasing enzyme. This suggests that the currently attributed AA matrix values for phytase may be overestimated. This study reinforces those concerns, emphasizing the need for more conservative AA matrix recommendations.

## Funding

This research received no grant from any funding agency or /sector.

## CRediT authorship contribution statement

**Cengizhan Mızrak:** Writing – review & editing, Writing – original draft, Visualization, Validation, Software, Resources, Methodology, Conceptualization. **Edvin Rey:** Writing – review & editing, Writing – original draft, Visualization, Validation, Supervision, Software, Resources, Project administration, Methodology, Investigation, Formal analysis, Data curation, Conceptualization. **Yusuf Cufadar:** Writing – review & editing, Writing – original draft, Visualization, Validation, Software, Resources, Project administration, Methodology, Investigation, Formal analysis, Data curation, Conceptualization.

## Disclosures

The authors declare no conflicts of interest.
